# Buzzwords in Females’ Ears? The Use of Buzz Songs in the Communication of Nightingales (*Luscinia megarhynchos*)

**DOI:** 10.1371/journal.pone.0045057

**Published:** 2012-09-13

**Authors:** Michael Weiss, Sarah Kiefer, Silke Kipper

**Affiliations:** Institute of Biology, Behavioural Biology Group, Free University Berlin, Berlin, Germany; University of Melbourne, Australia

## Abstract

Differences in individual male birds’ singing may serve as honest indicators of male quality in male-male competition and female mate choice. This has been shown e.g. for overall song output and repertoire size in many bird species. More recently, differences in structural song characteristics such as the performance of physically challenging song components were analysed in this regard. Here we show that buzz elements in the song of nightingales (*Luscinia megarhynchos*) hold the potential to serve as indicators of male quality and may therefore serve a communicative function. Buzzes were produced with considerable differences between males. The body weight of the males was correlated with one measure of these buzzes, namely the repetition rate of the buzz subunits, and individuals with larger repertoires sang buzzes at higher subunit-rates. A model of buzz performance constraints suggested that buzzes were sung with different proficiencies. In playback experiments, female nightingales showed more active behaviour when hearing buzz songs. The results support the idea that performance differences in the acoustic fine structure of song components are used in the communication of a large repertoire species such as the nightingale.

## Introduction

The singing of male song birds serves the two main functions of territory defence against other males and the attraction of females. Individual differences in song characteristics often lead to differences in reproductive success, making song a sexually selected signal [1], [2]. The theory of sexual selection claims that signals must be subject to some constraint in order to serve as an honest indicator of quality [3], [4]. Bird song is a complex acoustic signal consisting of many components that have evolved and are maintained by sexual selection. The relative importance of these components for communication differs across bird species [5].

Many studies have shown that, irrespective of ‘song content’, song output measures affect female choice. Examples are the time spent singing, song rate or song length [6]–[8].

For a long time, researchers have focused on song repertoire size as the primary target of female choice. In some species repertoire size might be an indicator of male quality as supported by correlations of repertoire size and body measures [9]–[11] and volume of brain nuclei related to song [12]. Evidence for female preferences for large repertoires was often provided in lab-experiments but only in a few field studies [10], [13]–[15] and many studies failed to find such preferences [16], [17].

Some studies on quality-related song characteristics went a step further from focussing on repertoire size and investigated song production mechanisms, suggesting that singing some specific song structures might be physically more challenging than others. For example, such song structures might be produced in a way more demanding in metabolic and syringeal muscle activity than other song components [18]–[22]. Accordingly, such structural song characteristics have been suggested to serve as ‘index signals’ that honestly communicate a physical trait related to the song performance [23].

So far, mostly syllables that are rapidly repeated in trills were investigated in this regard [24]–[27]. In these studies, the performance limit of song production was described by the relationship between trill rate and frequency bandwidth. Other studies revealed correlations between aspects of male quality and the consistency of their trill performance [28], [29] or specific song structures such as rattles [30], snarrs [31] or buzzes [32]. Rattles, snarrs, and buzzes share certain characteristics such as a large frequency bandwidth, a ‘pulsed’ structure and a long duration (compared to other song elements). Since they differ considerably in other measures such as the mean frequency and pulse rate, their acoustic pattern does not necessarily suggest a common production mechanism. For buzzes, i.e. pulsed low-frequency elements with long duration, it has been suggested that in particular the duration of such vocal elements holds the potential to reflect performance trade-offs [19], [32].

Such structural song traits may be used to convey quality information predominantly in species with simply structured songs and small repertoires. However, even in large repertoire species, the performance of some song components might contain information different from (or in addition to) repertoire size [17]. In general, to investigate the potential of communicative signals to serve as an indicator of quality, it is necessary to show that a signal a) has large inter-individual variation; b) is reliably related to a physical trait; and c) is indeed used in communication by potential receivers. Here, we investigate whether a complex structural song trait, namely the buzz element, produced by male common nightingales, *Luscinia megarhynchos*, conforms to the above three predictions and is thus likely to serve as an acoustic signal of male quality.

Nightingales learn and sing their song types very precisely and stereotyped [33]–[35] and therefore allow reliable comparisons of song types within and across males. Nightingales have extraordinarily large song type repertoires (approx. 180 different song types per male) and repertoire size has been suggested to be an honest indicator of male quality since it is correlated with male qualities such as body measures and arrival date at the breeding site and with age [11], [36], [37]. Though the production and performance patterns of individual’s song structures beyond repertoire composition have not been investigated in this species so far, there exist at least some indirect hints that specific structures in the song might reflect performance limits. For example, in simulated male-male interactions, nightingales showed stronger responses to playbacks containing many broadband trill songs and males that successfully paired later in the season responded more strongly to trills than males that remained unpaired [38], [39]. Similarly, whistle songs have been suggested to reflect individual characteristics of males potentially related to quality [40]. However, subtle intra- and inter-individual differences in song production of specific song elements have not gained that much attention so far.

Here we investigate one song element in the singing of nightingales under this regard, namely buzz elements. Buzzes are acoustically and syntactically peculiar elements in nightingale songs which are produced by very fast repetitions of sound subunits in a narrow and rather low frequency range. Buzzes occur in several song types and the performance of buzzes might be a good indicator of male quality. We investigated whether buzzes hold the potential to serve as index signals in nightingales by testing three predictions. Firstly, we hypothesized that buzzes show larger variation between individuals than within individuals. Secondly, we expected that the acoustic parameters of buzzes are related to quality measures of individuals such as age, breeding stage or body measures. In addition, we tested whether buzz acoustics are related to repertoire size. Thirdly, to investigate whether buzzes function in communication we tested whether male and female nightingales react differently when hearing songs containing or not containing buzzes.

## Materials and Methods

### Study Population

The study was conducted on a population of nightingales in Berlin - Treptower Park, a municipal city park [38]. As part of a long-term project on song and breeding behaviour, males of the population were individually marked by coloured leg rings.

### Song Recording and Analysis

Nocturnal song was recorded using a Sennheiser ME66/K6 directional microphone connected to a portable Marantz PMD-600 solid state recorder or a Sony TCD 5 tape recorder. All sound analyses were conducted with the software Avisoft SASlab Pro 4.52 (R. Specht, Berlin, Germany).

We analyzed nocturnal song of 59 males (1 hr of spontaneous song of each male from yrs. 2003–2010, each male contributed only once). We used these analyses to determine the population repertoire of buzz song types and to determine how often the different buzz song types were performed per bird. Further analyses were conducted with subsets of this sample and additional birds (details in respective sections). The selection of these subsets based in all cases exclusively on the availability of suitable recordings for the respective analyses or comparisons (e.g. early-late season or successive years). All statistical analyses and tests were selected based on the data structure (e.g., scaling or distribution) to meet test assumptions and were calculated with R 2.9.1 [41].

**Figure 1:**
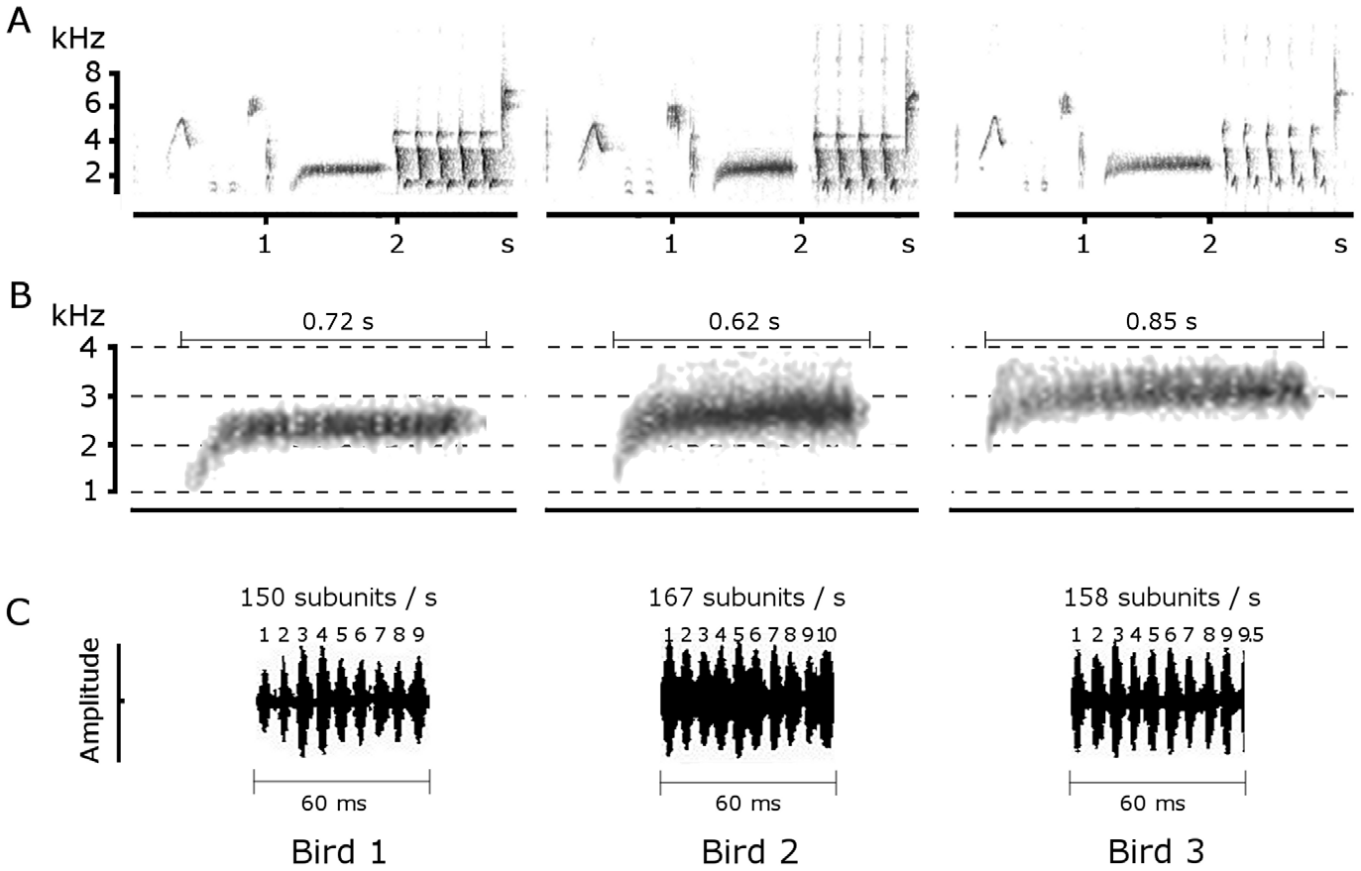
Buzz description. **A** Buzz songs of the same type produced by three different individuals. A buzz is a long, narrow element produced with a rather low frequency. Even though songs and buzzes appear very similar at a first look, acoustic characteristics differ between males. **B** The same buzz elements depicted with enlarged scale, showing that buzzes were produced with different mean frequencies and durations. **C** Amplitude envelope curve of subsections of the buzzes. The small subunits are clearly visible. Again, the buzz rates are individually different.

Visual comparisons and preliminary acoustic analyses suggested three measures to be most decisive in describing buzzes: the length of the buzz (Figure 1A and 1B), the mean frequency of the buzz, (measured as the frequency with the highest amplitude averaged over the whole buzz element, Figure 1B), and the rate per second of the small subunits of the buzz (determined by zooming into the amplitude envelope curve; Figure 1C). In the following we define this rate of subunits per second as buzz rate.

### Analysis of Buzz Song Characteristics

To investigate individual differences in buzz production, we analysed buzzes produced within the four most common buzz song types (most common across the repertoires of all individuals) in 1 hr nocturnal song of 15 male nightingales. These males were selected randomly based solely on the quality of the song recording (the buzz rate estimation in particular required recordings with excellent signal-to-noise ratio). Given that all recordings were obtained during the first days of the breeding season and males still sang at night (which they mostly stop after pair formation [42]) makes it most probable that these males were (still) unmated and represent a good cross section of the population under study.

### Investigation of Inter-individual Variation in Buzz Production

For each buzz we measured duration, mean frequency, and buzz rate. To analyse individual differences (and in all other analyses of this study), only buzzes of the same buzz song type were compared, because a preliminary analysis revealed that buzzes in different song types were produced with significant different durations and mean frequencies (ANOVA: n = 15, duration: F(3,153) = 8.9, p<0.001, frequency: F(3,153) = 752.7, p<0.001, buzz rate: F(3,153) = 2.64, p = 0.051). We characterised songs as belonging to the same song type when they differed in not more than three of approximately ten element types in the first two sections of the song and included the same repetitive sections [35], [43].

To compare the individual variation in the singing of buzz elements with that of other elements we additionally measured the elements sang immediately before and after the buzz in the song type shown in Figure 1. Since other elements do not possess subunits, we restricted our analysis to the two other measures: mean frequency of element (measured as the frequency with the highest amplitude averaged over the whole element) and duration. To account for the different mean durations and frequencies of the three elements we used coefficients of variation as described in e.g. [44]. We used the singing of the same 15 individuals for this analyses that were used in the analysis of buzz element variation. Three birds didn’t sing the respective song type, and one bird had to be excluded from the analysis because it sang a different element type before the buzz element. We thus included 41 versions of the same buzz song type sang by 11 individuals with 2–8 versions per individual (median 3) in the analysis. We calculated coefficients of variation per element and individual. The data from the 15 males were used to explore possible performance constraints on buzz rate. First, we analysed intra- and inter-individual variance of the three measures. In the singing of individual males, for each of the different song types containing buzzes, individual’s buzz rate and mean frequency were rather stable whereas the duration of the buzzes turned out to be the most variable buzz component (mean variance of standardised buzz measures (z-scores): duration = 0.25, buzz rate = 0.15 and mean frequency = 0.02, n = 15). These differences were significant: n = 15 males, with stratification by 4 buzz types, with variable numbers of buzzes per type and male, maxT = 3.17, p- = 0.004 (Independence test as implemented in the R package ‘coin’ [45], [46]. In addition, measuring buzzes had shown that, in the whole buzz, the mean frequency and the buzz rate are stable, whereas the duration remains the only variable component. Given our results and those of similar studies exploring production mechanisms of buzz-like structures e.g. [19] we then analyzed the effects of buzz rate and mean frequency (and possible interactions) on buzz duration using a linear mixed-effect model (LMM) with individual subject fitted as random factor to account for the repeated sampling of the same individuals [47], [48].

Based on the relationships of buzz rate, frequency and duration as described above we calculated a regular regression of buzz rate and frequency on duration. Considering that a regular regression might not be the only way to analyse these relationships, we also provide data on a three dimensional upper-bound regression comparable to the upper-bound regression reflecting the two-dimensional performance constraint of trill rates and frequency bandwidth in [24]. A visual inspection of the bi-variate relationships of buzz rate and duration and frequency and duration suggested a triangular distribution of data points and a distinct upper bound and justified this approach. For a comparison of both approaches to describe performance constraints see [49].

**Figure 2:**
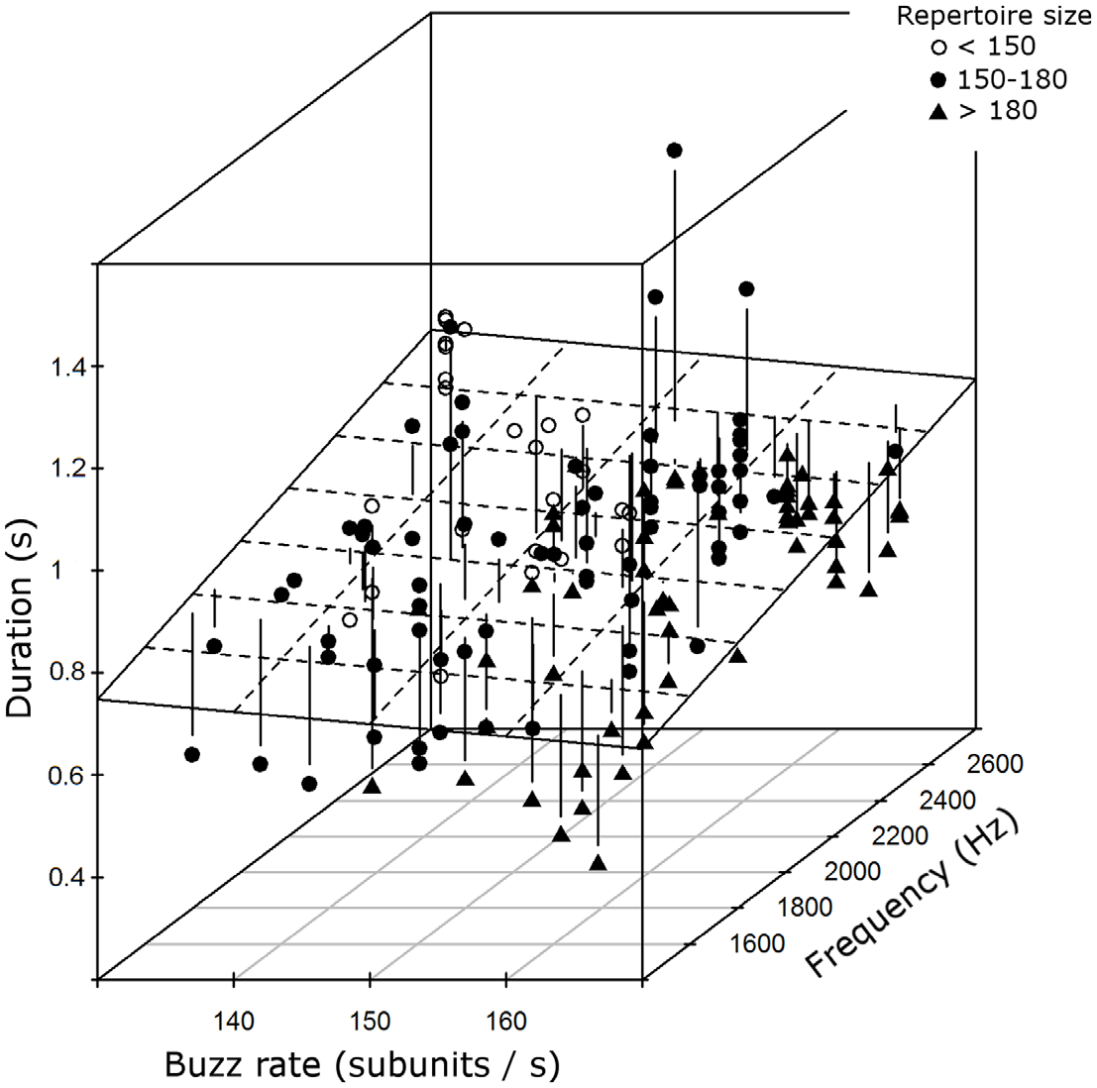
Model of buzz constraints. Buzz rate, mean frequency and duration of 157 buzzes produced by 15 individuals. The surface shows the upper bound regression calculated by a subset of these data (see methods). Vertical lines reflect the distance of the actual buzz duration to the length predicted by the upper bound regression (residuals of duration to the upper bound regression). The 15 birds are clustered according to their repertoire size in three classes (open circles = birds with small repertoires (<150 song types, n = 3). Filled circles = birds with medium size repertoires (n = 7) and filled triangles = birds with large repertoires (>180 song types, n = 5). See text for further explanations.

According to the method used in [24] and [50] we separated the mean frequencies and buzz rates into distinct categories and identified for each combination of these categories the longest buzz and calculated the upper-bound regression by using these data exclusively resulting in the surface shown in Figure 2. Bin categories for the upper bound regression were 8 bins for mean frequency (bin size 160 Hz) and 15 bins for rate (bin size 3 subunits/s).

### Buzzes and Male Quality Measures

To explore whether buzz production was under some form of physiological constraint and thus might act as an honest indicator of male quality, we analysed individuals’ buzz rates, buzz mean frequencies and durations and their buzz performance (measured as the distances of individuals’ buzzes to the performance limit suggested by the upper bound regression) in relation to individuals’ repertoire sizes. Repertoire size for the birds in our sample was determined as the number of different song types in 533 consecutive songs (approximately 1 hr spontaneous nocturnal singing), following [11]. This sample size has been shown to be sufficient to approach the asymptote of repertoire curves in nightingales [36].

We investigated correlations of repertoire size and buzz production by using a generalised linear model (GLM) fitting the effects of buzz rate, mean frequency and buzz length (mean per bird each) to repertoire size with Poisson error distribution [47], [48].

For 14 nightingales we compared buzz characteristics and body measures. In spring 2010, nightingales were trapped using Ecotone mist nets (http://www.ecotone.pl), sexed and aged (1 year or older; only birds older than one were included in this analysis), and measured (length of tarsus (±0.1 mm), wing length (±0.5 mm), and body mass (±0.1 g)). The age of the nightingales was determined by characteristic feather features [51]–[53]. We used Spearman rank- and Pearsons correlation to analyse correlations of body measures and buzz measures (average of the buzz measures per bird).

To investigate changes in buzz production in the course of the singing season, we compared the singing of nine birds (for which recordings were available) in the first phase of the breeding season (April or first week in May, i.e. shortly after arrival) and later in the season (second half of May or beginning of June) with at least 3 weeks between the two recordings. Since nightingales usually cease nocturnal singing after pairing [42]; these males had most probably remained unmated. If singing in late season is still aiming at attracting females, we would expect males to sing buzzes of same or even higher quality than early in the season. However, since late pairings are extremely rare and nightingales are breeding obligatory only once per season (not leaving possibilities for extra-pair mating in later breeding attempts), nocturnal singing late in the season might be for other reason including advertising quality for future seasons e.g. [54]. If that would be the case, song motivation might have decreased and flexible buzz measures might show a decrease. Again, only the same buzz song types produced by the same individual early and late in the season were compared (see above). We used paired t-tests and exact paired Wilcoxon signed rank tests to test for changes in buzz rate, mean frequency and buzz duration [55].

In order to analyse changes in buzz production across years we compared the singing of eight nightingales in two successive years, again only including birds that were at least two years old in the first year of analysis and only using recordings from the beginning of the season. Again, only the same buzz song types produced by the same individual in both years were compared (see above). We used paired t-test and exact paired Wilcoxon signed rank test for statistical analysis. The singing of eight nightingales in their first and second breeding season was compared in the same way in order to investigate whether and how these age classes differ in buzzing. Previous studies had shown that the repertoire of one year old nightingales differed considerably from that of older birds, with repertoires being about a third smaller in younger birds [36], [37].

### Do Buzzes Function in Communication? Playback Experiments with Males and Females

Our investigations whether buzzes yield the properties to serve as a quality indicating signal were paralleled by playback experiments addressing reactions of male and female nightingales to buzz songs. Each bird was tested with two playbacks: a ‘buzz playback’ (3 to 6 buzz songs in 20 songs) and a ‘non buzz playback’ (0 buzz songs in 20 songs). We used recordings of 12 different nightingales unknown to the focus birds (warranted by distance in time and space) as sources for the playback stimuli. From each source bird we randomly chose 40 different song types including some buzz song types. Each bird received two playbacks consisting of the song of one source bird with at least one hour pause between playbacks. One playback was composed of 20 songs contained 3 to 6 buzz songs and one playback of 20 different songs without buzz songs. Sequence of treatments was randomized across birds. The playback files were broadcasted with a portable MP X10i, ODYS player in.wav-format. The player was connected to a custom-build speaker (DKA Daniel Kiefer Audio, Heidelberg, Germany, for details see [56] constructed under consideration of the suggestions in [57].

Twelve spontaneously singing male nightingales in Berlin Treptower Park were tested at night with interactive playbacks (each playback song was started after the focus bird had finished its own song) in spring 2007 and 2008 (25 April and 6 May, between 0000 and 0200 hours). Songs were broadcast from at least 15 m away from the singing bird (for more details see [58]). Playback volume was standardized to peak amplitude of 86 dB SPL at 1 m distance (as measured with a CEL 314 precision sound level metre, integration time 125 ms). This corresponds to natural amplitude peaks measured in singing males [59].

Numerous studies had shown that nightingales readily respond to nocturnal playbacks and adjust their singing depending on the playback stimuli e.g. [56], [58], reviewed in [60]. As response measures we analysed the number of buzz songs sang during and after the playback as well as song rate, song length, number of song type matches and percent of playback songs overlapped by the bird during the playback. We measured 2 minutes of birds’ song before and after the playback (a duration comparable to the playback length). For the response measures song length and song rate we related the values to birds singing before playback onset by calculating differences of the values during and after the playback to the values before playback onset. For all other measures we compared the absolute values of the two different treatments.

In addition, we tested six hand raised female nightingales in the lab in late spring 2008 (19–20 June) in early morning (0600–0800 hours) with the same playbacks. Three of the six females received estradiol implants to increase reactivity 15 days before the playback experiments; the other females were sham-implanted. The pellets were made by mixing crystalline 17-beta-Estradiol with medical grade Silastic adhesive (Dow-Corning) in a ratio of 1∶8 and extruding this mixture as a thin rope through syringe. This mixture was cured by drying overnight, then weighted and cut into pellets of about 5 mm lengths. Pellets were implanted under the skin covering the breast muscle. The control group was similarly implanted with rope pellets composed of pure Silastic adhesive without estradiol (for similar methods and more details see e.g. [6].

Females were kept single-wise in plastic cages (50*120*50 cm) under weekly changing light conditions reflecting the natural conditions during the breeding season (in the week of experiments: lights on 0500–2130 hours). The bottom of the cage was covered with paper and grit and cages contained 6 perches. Under neutral conditions, perches and bottom were regularly used by females.

Since hormone treatment did (in this and similar experiments) not seem to affect female behaviour in response to song we decided to pool results. Playbacks were presented non-interactively with standardised pauses of 3.5 s between songs and one hour pause between the two treatments. Since females did not perform unambiguous copulation solicitation displays (CSD), we decided to instead assess the general behavioural activity of the females by counting the number of location changes and the number of tail lifts the females performed while listening to a playback as measures of general arousal. Similar response measures were used in other studies on female song preferences [61], [62]. For statistical analyses we calculated exact paired Wilcoxon signed rank tests.

## Results

### Intra- and Inter-individual Variation in Buzzes

The population repertoire of buzz song types (n = 59 males) consisted of 13 different types in altogether 425 distinct song types. Individuals sang 2 to 7 different buzz song types (median = 5) and 8 to 27 buzz songs per hour (median = 14). There was no correlation between the repertoire sizes of individuals and neither the number of buzz songs nor the number of different buzz songs sang in 1 hr spontaneous nocturnal singing.

Comparing buzz characteristics across 15 individuals revealed that males differed remarkably in the acoustic fine structure of their buzzes. In all of the four most common buzz song types, the three buzz characteristics measured (buzz rate, mean frequency and duration) differed significantly between individuals (ANOVA: buzz rate: F(14,142) = 25.13, p<0.001, mean frequency: F(14,142) = 3.36, p<0.001, duration: F(14,142) = 11.22, p<0.001). To test whether these differences were larger than differences in other elements of the song, we also measured the elements immediately preceding and following the buzz in one song type (see Fig. 1A for illustration). The variation within buzz element duration was significantly higher than the variation within the length of both other elements (Table 1). In contrast, there was no significant different variation in the duration of the element before and after the buzz (Table 1). To support these findings we also explored the variation in the mean frequency of the three elements the same way. The variation in the mean frequency of the element preceding and following the buzz were both lower, although the latter not significantly so, than that of the buzz element (Table 1). The variations of mean frequencies of the element before and after the buzz were comparable (Table 1).

**Table 1:** Variation in buzz elements is higher than in other elements.

		*estimate*	*se*	*t*	*p*
duration	buzz-prebuzz	−0.045	0.016	−2.78	**0.012**
	buzz-postbuzz	−0.069	0.016	−4.28	**<0.001**
	prebuzz-postbuzz	−0.024	0.016	−1.49	0.15
meanfrequency	buzz-prebuzz	−0.008	0.003	−2.40	**0.026**
	buzz-postbuzz	−0.006	0.003	−1.60	0.12
	prebuzz-postbuzz	0.003	0.003	0.90	0.43

Results of linear mixed effect model analyses with variation coefficient of element duration and mean frequency as response measures and element type as fixed factor and bird as random factor (to account for repeated measurement of elements per individual), n = 11.

A global comparison of the three buzz measures between individual males indicated that males that produced very long buzzes sang these buzzes at rather slow buzz rates and with relatively high frequencies. On the other hand, the higher the buzz rate and the lower the mean frequency of the buzz, the shorter were the buzzes. Altogether the interrelation of the three measures suggested a production constraint including all three variables.

A linear mixed effect model analysis revealed a significant negative effect of buzz rate and a significant positive effect of mean frequency on buzz duration (df = 136, buzz rate: t = −3.74, p<0.001, mean frequency: t = 2.1, p = 0.037). The significant effect of the interaction term of buzz rate and mean frequency on the length of the buzz element in the full model indicates the interdependency of both effects (df = 136, buzz rate x mean frequency: t = −3.46, p<0.001, song type was additionally used as fixed factor (t = 2.08, p = 0.038) and individual birds as random factor).

Figure 2 presents this complex subject graphically. Due to the repeated measurement and the resulting error structure it was not possible to depict a regression plane from the above model in a meaningful way. Instead of this we show a regression plane calculated from an upper regression. Thus, the surface in Figure 2 depicts the performance limit of buzz durations at different regions of the buzz rate - mean frequency space as calculated by an upper bound regression of a subset of data (see methods) (df = 49, buzz rate: t = −0.84, p = 0.41, mean frequency, t = 2.14, p = 0.038) Though a part of this analysis failed to reach significance (probably due to bin sizes selected), the surface nevertheless reflects a potential performance limit. To give an example: only a buzz with a short duration appears to be producible with many subunits and low frequencies.

In general, individual birds did not differ in their overall vertical distance from the upper bound regression (ANOVA, with vertical distance as response and individual birds as factor, F(14,142) = 0.39, p = 0.54) and repertoire size was not correlated with this distance (Spearman rank correlation, r = −0.06, p = 0.41). Looking in more detail though, individual birds (and in particular birds with different repertoire sizes) differed in their buzz production in terms of the position of their buzzes in the buzz rate - mean frequency space (Figure 2). Individuals with large repertoires produced shorter buzzes than individuals with small repertoires, but they performed their buzzes at other regions of the buzz rate - mean frequency space. The right half of this space is reserved for individuals with medium and large repertoire sizes and the right front region with very high buzz rates and very low mean frequencies at the same time holds exclusively individuals with very large repertoires (Figure 2). This is furthermore corroborated by a significant positive effect of buzz rate (mean per bird) on repertoire size (GLM: df = 14, buzz rate: z = 3.46, p<0.001, mean frequency: z = −0.44, p = 0.66, duration: z = −1.18, p = 0.24).

### Buzzes and Quality Measures

**Figure 3:**
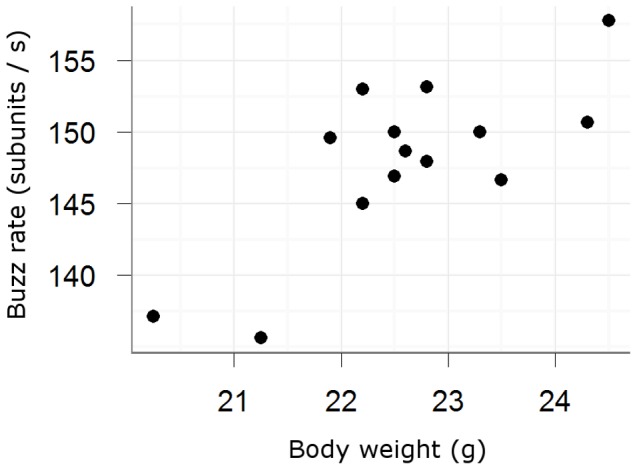
Correlation of body weight and buzz rate. Correlation of body weight and buzz rate of 14 male nightingales. Pearson correlation: n = 14, r = 0.76, p = 0.002.

In order to correlate buzz characteristics with body measures, we analysed the four most common buzz song types produced by each of 14 individuals. Values used in this calculation were mean duration, mean frequency and mean buzz rate per bird. The weight of the birds was positively correlated with buzz rate (Pearson correlation: n = 14, r = 0.76, p = 0.002, Figure 3). No significant correlation was found between wing and tarsus length and buzz rate as well as between buzz duration and mean frequency and any of the body measures (n = 14, all p-values >0.28).

The comparison of buzzes produced early versus late within a breeding season revealed that nightingales produced significantly shorter buzzes late in the season (paired t-test: n = 9, t = 3.82, p<0.001). Additionally, in the late phase of the season buzzes were produced with lower mean frequencies and lower buzz rates (mean frequencies: paired t-test: n = 9, t = 3.62, p<0.001; buzz rates: exact paired Wilcoxon signed rank test: n = 9, W = 37.5, p = 0.005) and nightingales sang significantly more buzz songs late in the season (n = 9, W = 1.5, p = 0.023).

We found no significant differences in buzzes produced by the same individual in the early phase of two subsequent breeding seasons when birds were at least two years old when first recorded (buzz rate: exact paired Wilcoxon signed rank test: n = 8, W = 134.5, p = 0.30, mean frequency: paired t-test: n = 8, t = −1.65, p = 0.11, duration: paired t-test: n = 8, t = −0.31 p = 0.76).

In another longitudinal analysis we compared buzz characteristics of 8 nightingales in their first and second breeding season (birds were one year old when first recorded). We did not find any significant differences. That is, in contrast to repertoire size, neither buzz rate (exact paired Wilcoxon signed rank test: n = 8, W = 93.5, p = 0.18) nor mean frequency (paired t-test: n = 8, t = 0.99, p = 0.33) nor buzz length (paired t-test: n = 8, t = −0.29, p = 0.77) differed between the first and second breeding season of individuals.

### Responses to Buzzes in Playback Experiments

In a nocturnal playback, male nightingales did not change their song characteristics significantly different in response to playbacks with or without buzz songs (all n = 12, all p>0.05 for all response variables: no. of buzzes during and after playback, song rate, no. of song type matches, percentage of songs overlapped in exact paired Wilcoxon signed rank and mean song length in paired t-tests). In both playback treatments the birds sang significantly shorter songs after playback onset compared to their singing before the playback (paired t.test: n = 12, treatment buzz: t = −3.83, p = 0.003, treatment without buzz: t = −4.45, p = 0.001), indicating that birds’ singing was in general affected by the playback, but males did not differentiate between the two treatments.

**Figure 4:**
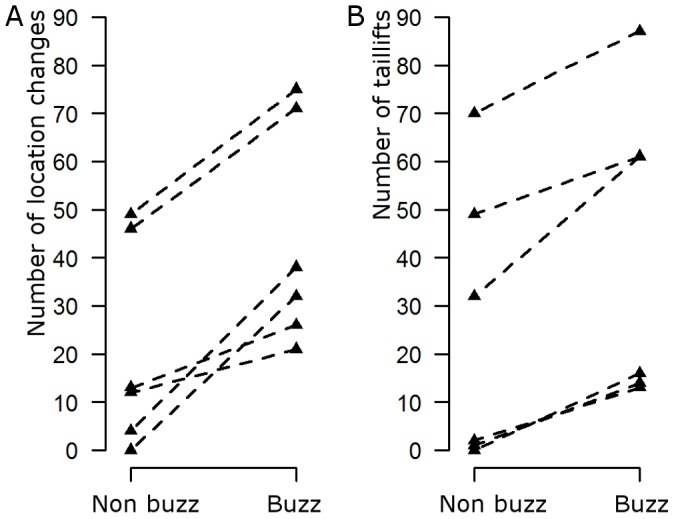
Playback experiment. Responses of six female nightingales to playbacks containing or not containing buzz songs. **A** the number of location changes and **B** the number of tail lifts the females performed during the playbacks. For both responses, exact paired Wilcoxon signed rank test, p<0.05.

In contrast, female nightingales showed more active behaviour while listening to the buzz playback than when listening to the non-buzz playback (Figure 4). The number of location changes was higher in all six females while being exposed to the buzz playback as compared to the playback without buzzes, as was the number of tail lifts (Figure 4, exact paired Wilcoxon signed rank test: location changes: n = 6, W = 21, p = 0.031, tail lifts: n = 6, W = 21, p = 0.031).

## Discussion

Nightingales sang their buzzes with considerable individual differences in buzz rates, mean frequencies as well as durations. One of the buzz measures was correlated with repertoire size: males with larger repertoires sang buzzes with higher rates. Furthermore heavier individuals produced buzzes with higher repetition rates. Female nightingales responded with increased activity when hearing a buzz playback. To conclude, buzzes fulfil many preconditions to serve as an indicator of individual quality in nightingale communication.

Following the idea that buzzes might be produced under performance trade-offs and based on the results of regression analyses we suggest the following explanatory scheme that includes the three buzz measures simultaneously and relates potential buzz performance trade-offs to other characteristics of the signaller as its overall repertoire size. Our results suggest that buzzes with high rates and low frequencies might be more challenging to produce and these two characteristics have strong effects for the duration of the buzz as the most flexible buzz measure. Following our scheme, a buzz of very ‘high quality’ would have a high buzz rate and at the same time a low mean frequency and is produced for as long as possible under these conditions. Though the production mechanism of buzzes in nightingales has not yet been investigated, insights on production mechanisms of similar low-frequency, broad-band sounds fit very well with the performance trade-offs our analysis suggests: A buzz is most likely produced during a single, pulsatile expiration and the duration might thus be limited [19]. The buzz rate might be the result of syringeal muscle activity, as has been shown for similar low-frequency sounds in starlings [63] and thrashers [64]. The high buzz rates we measured might reflect the upper limit of the muscular activity rate. Alternatively, a buzz structure might be produced without direct muscular control of the buzz rate by vibration of syringeal structures [65], but even in this case, the rate and duration might reflect individual performance trade-offs.

The acoustic structure of buzzes produced by the same individual early and late in a breeding season differed. Buzzes sung late in the season were shorter, of lower frequency, and lower buzz rate. It is important here to consider that this result does not necessarily reflect the general change in buzz production over the season across all males since we only included males that continued nocturnal song (thus presumably not paired, thus presumably of ‘lower quality’). However, our results hint to a context-dependent intra-individual plasticity in buzz production. This suggests that buzzes are not learned once and later produced by individual nightingales at determined rates, frequencies and durations. This might either reflect a change in the male characteristics being signalled by the buzzes (e.g. change in body mass throughout the season) or, alternatively, the buzz performance might be context dependent and modifiable within limits and thereby might deliver respective information about the singer. The decrease in buzz rate and length in later phases of the breeding season might correspond to decreasing motivation of the singers. This result might appear contradictory to the idea of ‘index signals’. But then, it has been suggested for long that bird song structures might encode multiple information [66], reviewed in [5]. Recent studies confirmed that even index signals such as vocal trill performance might be modulated in different contexts within narrow limits [27]. Nonetheless our results suggest the existence of a robust individual limit of buzz performance, because individuals did not change their buzz performance significantly between different breeding seasons, when buzzes produced early in both seasons (probably in a highly motivated state of all singers) were compared. If the above holds, then buzz performance may be used in addition to repertoire size to assess signallers’ general quality and additionally might provide dynamic information about short term changes in their condition or motivation.

The singing responses of males in response to playbacks with or without buzzes did not differ. Female nightingales though showed more active behaviour during a playback containing buzz songs compared to a playback without buzz songs. Along with the correlative findings of buzz performance and male body condition, this supports the notion that buzzes play a role in nightingale communication. Our experiments were unable to determine whether females differentiate between buzzes of different quality or whether the patterns we observed for buzzes can be generalized. Interestingly, a recent study in banded wrens found that rattle and buzz elements were used mostly in male-male counter-singing and were less likely to be produced in the presence of a female [32], suggesting that apparently similar acoustic structures may indeed be used in different contexts in other species. However, assuming that other song elements display similar individual differences, and are thus information-encoding, our data support the idea that assessment of structural differences within the ‘same’ song types may be more biologically relevant than simply investigating repertoire composition [67]. Our study contributes to the growing body of evidence that in addition to the song type level (macro-structure such as repertoire size or syntax-like rules of song sequences), the micro-structure of song with performance measures might as well encode important information.
